# *Galleria mellonella *in vitro model for chromoblastomycosis shows large differences in virulence between isolates

**DOI:** 10.1186/s43008-023-00134-5

**Published:** 2024-03-08

**Authors:** Dongmei Shi, Zhiya Yang, Wanqing Liao, Chen Liu, Liang Zhao, Huilin Su, Xiaodong Wang, Huan Mei, Min Chen, Yinggai Song, Sybren de Hoog, Shuwen Deng

**Affiliations:** 1 Department of Dermatology & Laboratory of Medical Mycology, Jining No. 1 People’s Hospital, Shandong, China; 2 The Laboratory of Medical Mycology, Jining No. 1 People’s Hospital, Shandong, China; 3 Department of Dermatology, Shanghai Key Laboratory of Molecular Medical Mycology, Changzheng Hospital, Shanghai, China; 4 Key Laboratory of Environmental Pollution Monitoring and Disease Control, Ministry of Education of Guizhou & Guizhou Talent Base for Microbiology and Human Health, School of Basic Medical Sciences, Guizhou Medical University, Guiyang, China; 5 Department of Dermatology, The First Affiliated Hospital of Sun Yat-Sen University, Guangzhou, China; 6 Department of Dermatology, the First Affiliated Hospital of Xinjiang Medical University, Urumqi, China; 7 Institute of Dermatology, Chinese Academy of Medical Science, Nanjing, China; 8 Department of Dermatology and Venereology, First Hospital in Peking University, Beijing, China; 9 Center of Expertise in Mycology of Radboud University Medical Center, Canisius Wilhelmina Hospital, Nijmegen, The Netherlands; 10 Department of Medical Microbiology, The People’s Hospital of Suzhou New District, Suzhou, Jiangsu China

**Keywords:** *Galleria mellonella*, Chromoblastomycosis, *Cladophialophora carrionii*, Virulence, Antifungal efficacy in vivo

## Abstract

**Background:**

Chromoblastomycosis is the World Health Organization (WHO)-recognized fungal implantation disease that eventually leads to severe mutilation. *Cladophialophora carrionii* (*C. carrionii*) is one of the agents. However, the pathogenesis of *C. carrionii* is not fully investigated yet.

**Methods:**

We investigated the pathogenic potential of the fungus in a *Galleria mellonella (G. mellonella*) larvae infection model. Six strains of *C. carrionii,* and three of its environmental relative *C. yegresii* were tested. The *G. mellonella* model was also applied to determine antifungal efficacy of amphotericin B, itraconazole, voriconazole, posaconazole, and terbinafine.

**Results:**

All strains were able to infect the larvae, but virulence potentials were strain-specific and showed no correlation with clinical background of the respective isolate. Survival of larvae also varied with infection dose, and with growth speed and melanization of the fungus. Posaconazole and voriconazole exhibited best activity against *Cladophialophora*, followed by itraconazole and terbinafine, while limited efficacy was seen for amphotericin B.

**Conclusion:**

Infection behavior deviates significantly between strains. In vitro antifungal susceptibility of tested strains only partly explained the limited treatment efficacy in vivo.

**Supplementary Information:**

The online version contains supplementary material available at 10.1186/s43008-023-00134-5.

## Introduction

Chromoblastomycosis (CBM) is a chronic, severely mutilating fungal skin disease with hyperendemicity in Brazil, China and Madagascar (Queiroz Telles et al. 2017). Patients develop dry, expanding, acanthotic lesions, where the fungus is present in the form of spherical, melanized “muriform cells”. A wide diversity of clinical types is known, with or without expansion of host tissue, but invariably lacking the tissue necrosis that characterizes phaeohyphomycosis. Without appropriate long-term therapy, the disease continues, frequently with satellites elsewhere on the body resulting from self-inoculation by scratching (Shi et al. 2016). CBM is regarded to be an implantation disease, i.e., an infection after traumatic inoculation of the fungus from an environmental source (Queiroz Telles et al. 2017), and is prevalent in rural populations in tropical and subtropical regions deprived from appropriate medical care. Etiologic agents are isolated from the environment with difficulty (Vicente et al. 2008), but metagenomics revealed their presence (Costa et al. 2020), suggesting that they have requirements for axenic growth differing from those of their strictly environmental counterparts that are isolated much easier (Vicente et al. 2014). Environmental occurrence of causative agents is also suggested by their differential prevalence under deviating climatic conditions. In humid, subtropical climate zones around the globe, *Fonsecaea* species are the prime cause of CBM, while in arid zones, *Cladophialophora* species are prevalent (Rasamoelina et al. 2020). Both Fonsecaea and *Cladophialophora* are members of the most advanced family (Quan et al. 2020) of *Chaetothyriales*, the *Herpotrichiellaceae*, comprising the black yeasts and relatives, which are characterized by a consistent degree of melanization of hyphae and yeast cells.

*Cladophialophora carrionii* (*C. carrionii*) is primarily known from CBM patients in desert-like areas, where cactus thorns are thought to be the prevalent source of inoculation (Zeppenfeldt et al. 1994). However, a close non-pathogenic relative, *Cladophialophora yegresii (C. yegresii),* was also found to inhabit thorns of a cactus fence surrounding the home of a CBM patient whose infection was caused by *C. carrionii* (de Hoog et al. 2007). Consequently, closely related *Cladophialophora* species seem to deviate significantly in infection potentials, but the pathogenesis of *C. carrionii* is not fully understood yet.

*Galleria mellonella (G. mellonella)* is a widely adopted insect model to investigate the virulence of a broad range of human pathogens (Perdoni et al. 2014). Its response to infections, managed exclusively by an innate immune response, is considered a useful model for understanding the first steps of human host–pathogen interactions (Trevijano-Contador et al. 2015). Until now, *G. mellonella,* as an invertebrate host has been never used to study the pathogenesis of *Cladophialophora* spp.

In the present study, we compare clinical and environmental strains of *C. carrionii* and its environmental relative, *C. yegresii* using physiological parameters, antifungal resistance, and pathogenicity with a *G. mellonella* infection model.

## Material and methods

### Strains

Nine strains of the CBM agent *C. carrionii* (clinical and environmental isolates) and its molecular relative *C. yegresii* (environmental isolates) were selected; metadata of the 9 strains are listed in Table 1. Strains were obtained from the reference collection of the Centraalbureau voor Schimmelcultures (CBS, housed at the Westerdijk Fungal Biodiversity Institute, Utrecht, The Netherlands). Identification of strains was confirmed by sequencing of the internal transcribed spacer regions of rDNA, as described previously (Deng et al. 2013).

**Table 1:** General information of the nine strains of *Cladophialophora* spp. tested in this study

Species	Isolate Nr	Source	Origin	Growth Temp. (°C )			MIC (mg/L)			
				Range/optimum	37 °C	AmB	ITR	VOR	POS	TER
*C. carrionii*	CBS 131838	Human CBM	China, Shandong	12–37/30	+	4	0.016	0.063	0.016	0.016
*C. carrionii*	CBS 114402	Human CBM	Venezuela, Falcón	9–37/30	+	2	0.125	0.25	0.031	0.063
*C. carrionii*	CBS 100434	Human	Madagascar	12–37/30	+	0.5	0.031	0.25	0.031	0.031
*C. carrionii*	CBS 117900	Human CBM	Venezuela, Falcón	15–37/27–30	+	1	0.063	1	0.063	0.125
*C. carrionii*	CBS 861.96	Dry plant debris	Venezuela, Falcón	9–37/27	+	8	0.031	0.5	0.031	0.063
*C. carrionii*	CBS 131736	Soil	Venezuela	9–37/30	+	4	0.031	0.125	0.063	0.25
*C. yegresii*	CBS 114405	Cactus	Venezuela, Falcón State	12–33/27	–	0.25	0.25	0.063	0.125	0.063
*C. yegresii*	CBS 114406	Cactus	Venezuela, Falcón State	12–33/27	–	0.25	0.5	0.125	0.125	0.063
*C. yegresii*	CBS 114407	Cactus	Venezuela, Falcón State	12–33/27	–	0.5	0. 25	0.125	0.125	0.063

*C. carrionii: Cladophialophora carrionii; C. yegresii: Cladophialophora yegresii;* Growth Temp: growth tempreture on Sabouraud’s Glucose Agar. MIC: Minimum inhibitory concentration; AMB: Amphotericin B; ITR: Itraconazole; VOR:Voriconazole; POS: Posaconazole; TER: Terbinafine

### Fungal physiology and melanization

Expansion growth was measured on Oatmeal agar (OA), 2% Malt Extract Agar (MEA) and Potato Dextrose agar (PDA), all obtained from Oxoid (Basingstoke, U.K.) in 90-mm culture plates at 25 °C for 2 weeks. Temperature relations were determined on Sabouraud’s Glucose Agar (SGA; Difco, Vianen, The Netherlands) in 90-mm culture plates at 12, 18, 24, 30, 36, and 42 °C; colony diameters were recorded weekly for 28 days. Degrees of melanization were determined in cultures grown on PDA plates at 25 °C for 10 days. Ten mL phosphate-buffered saline (PBS) was poured onto the growing colony, carefully scratched with a sterilized swab, and conidial suspensions were pipetted, filtered using a syringe containing glass wool, transferred to a fresh tube and adjusted to a final concentration of 5 × 10^6^ conidia/mL. Suspensions were measured spectrophotometrically at 405 nm to determine melanin contents.

### *Galleria mellonella* infection and survival assays

Final sixth instar *G. mellonella* larvae were acquired from Vellinga Voedseldieren (Ridderkerk, The Netherlands) and maintained at room temperature on wood shavings in the dark until use. Larvae were used within 2 days of receipt. Larvae of approximately 300–500 mg showing no discoloration were selected for the experiments. Fungal strains were subcultured on MEA at 25 °C for 5 days and conidia were harvested in phosphate-buffered saline (PBS). Filtered conidial suspensions were quantified with a Bürker-Türk hemocytometer. Groups of 15 larvae were inoculated with increasing conidial densities (1 × 10^4^, 1 × 10^5^ and 1 × 10^6^ conidia/larva) of each strain tested. Inoculation was performed by injecting 40 μL fungal suspension in the last left pro-leg with an insulin 29G U-100 needle (BD Diagnostics, Sparks, MD, U.S.A.). As controls, untouched larvae, larvae pricked with the needle and larvae injected with PBS were included. Larvae were checked daily for survival for 10 days in parallel at 27 °C and 37 °C. If during these 10 days larvae formed pupa, these individuals were removed from the experiment.

### Fungal morphology in hemolymph

Fungal morphology in the hemolymph of *G. mellonella* larvae was verified at different time points in the course of infection. As controls, larvae injected with PBS were included. Hemolymph was removed through an incision made in the last proleg at 24 h, 3*d*, 7*d* and 10*d* after inoculation and slides were made in Shear’s mounting fluid without pigments (de Hoog et al. 2020). Micrographs were taken with a Nikon Eclipse 80i microscope and DS Camera Head DS-Fi1/DS-5 m/DS-2Mv/DS-2MBW using NIS-Element freeware package (Nikon Europe, Badhoevedorp, The Netherlands). Dimensions were taken with the Nikon Eclipse 80i measurement module on slides and the mean and standard deviations were calculated from 40 to 50 conidia.

### Histopathology

At 24 h, 3*d*, 7*d* and 10*d* after inoculation, larvae were dissected and fixed in 10% buffered formalin. Since the larval exoskeleton is impenetrable to most fixative reagents, 100 μL of the 10% buffered formalin was injected into the larvae (Wayne 2017). After 24 h fixation, whole larvae were dissected longitudinally into two halves with a scalpel and fixed in 10% formalin for at least another 48 h (Perdoni et al. 2014). The two halves of larvae were routinely processed for histology. Sections were stained with Periodic Acid-Schiff (PAS). Micrographs were taken by using K-viewer 1.5.3.1setup (www.kfbio.cn).

### Antifungal susceptibility

Minimum inhibitory concentrations (MIC) of amphotericin B (AMB), itraconazole (ITZ), voriconazole (VOR), posaconazole (POS), and terbinafine (TER) were determined for all 9 strains tested according to CLSI M38-A3 as described previously (Deng et al. 2013). Two strains (CBS 117900 and CBS 114406) were selected to determine antifungal efficacy of AMB, ITZ, VOR, POS, and TER in vivo using the *G. mellonella* system. Larvae were infected each with 1 × 10^5^ conidia and injected with single dose of each antifungal agent, respectively (Additional file 2: Table S1). Each antifungal agent was administered twice at 2 h and 4 h post-infection by a separate injection. Untouched larvae, larvae injected twice with PBS (to rule out damage by double injection) and with the drug diluted in PBS (to check for toxicity effects of the respective drugs) served as controls. A different proleg was used for each injection, starting from the left last proleg and rotating left to right and moving proximally (i.e., injecting through the left last proleg, right last proleg, penultimate left proleg, and penultimate right proleg, as needed). Larvae were incubated at 37 °C to enhance comparison with vertebrate hosts. For each test group, 15 larvae were used, and experiments were repeated at least three times.

### Statistics

All experiments were performed independently at least in triplicate. Survival rates were evaluated by Kaplan–Meier survival curves and analyzed with the logrank (Mantel-Cox) test using GraphPad Prism software. Comparisons between groups were performed by one-way analysis of variance (ANOVA), Bonferroni test with correction for multiple comparisons, or Student test. Differences were considered significant at *p* ≤ 0.05. Statistical significance was determined by applying logrank (Mantel-Cox) test, comparing untreated groups with groups that received antifungals.

## Results

### Growth speed and temperature

All fungal strains showed slow expansion growth when cultivated on OA, PDA, and MEA, respectively, at 25 °C for 2 weeks. The strains of *C. yegresii* were nearly identical with 10 mm diam, while those of *C. carrionii* showed variation between 10 and 18 mm (Additional file 1: Fig. S1). Slowest growth was observed in environmental strain *C. carrionii* CBS 861.96, and fastest in environmental strain *C. carrionii* CBS 131736 on all three media. *C. carrionii* has optimum growth at 27–30 °C with a maximum at 37 °C; *C. yegresii* grows optimally at 27 °C and has a maximum at 33 °C. No growth occurs at 37 °C; this temperature is fungistatic as all isolates showed regrowth when placed at 24 °C (Table 1).

### Melanization measurement

**Fig. 1:**
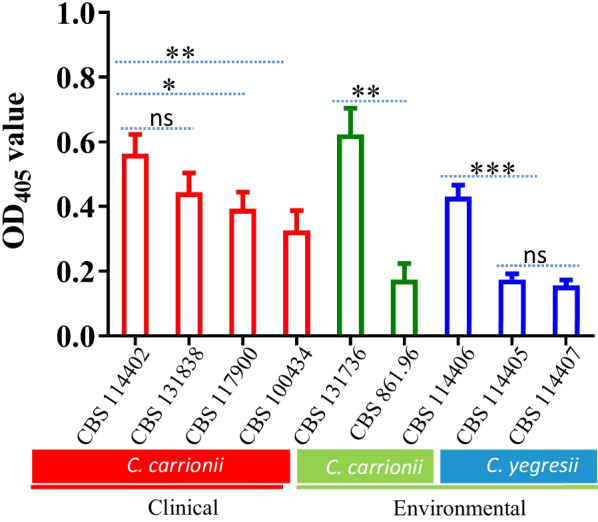
Melanin contents of studied strains of *Cladophialophora spp.* Strains cultured on PDA at 25 °C for 10 days. Optical densities of 10^6^/mL conidia suspensions were measured at 405 nm. *P* values shown with *p < 0.05, **p < 0.01, ***p < 0.005; ns, not significant

Melanization, measured at 405 nm after 10 days growth on PDA at 25 °C, varied significantly between strains (Fig. 1). Lowest degree of melanization was observed in environmental strain *C. carrionii* CBS 861.96, and highest in environmental strain *C. carrionii* CBS 131736 (*p* < 0.001). The two isolates of *C. yegresii* (CBS 114405 and CBS 114407) showed similar degrees of melanization (*p* > 0.05) but significant difference with CBS 114406 (*p* < 0.001) as observed in *C. carrionii*. On average, clinical strains of *C. carrionii* were slightly more melanized than environmental strains of *C. carrionii* and *C. yegresii* (Fig. 1).

### Survival curves

**Fig. 2:**
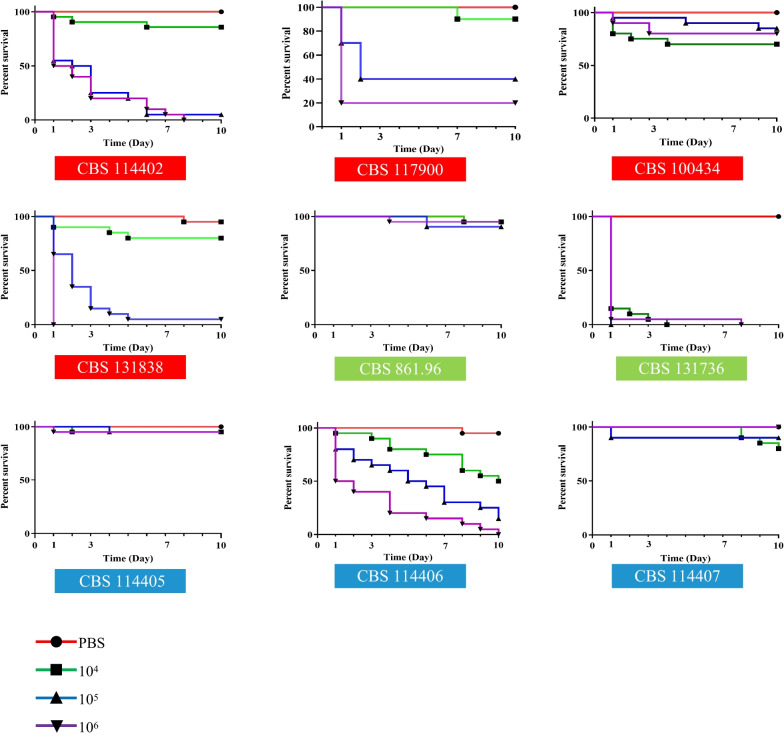
Strain and dose-dependent killing of *Galleria mellonella* larvae by *Cladophialophora carrionii* (red = clinical, green = environmental) and *Cladophialophora Yegresii* (blue) at 10^4^, 10^5^ and 10^6^ conidia/larve in *G. mellonella* and kept at 37 °C for up to 10 days. Curves were plotted from single experiment using 15 larvae

Killing rates of the nine strains selected in this study was dose-dependent of fungal inocula, regardless whether strains were clinical or environmental, and regardless of species affiliations (Fig. 2). For each experiment three different controls were used, and high survival was obtained for all control groups (untouched larvae, larvae only pricked by the needle and larvae injected with PBS; data not shown). In general, the 10^5^ and 10^6^ inocula resulted in 80–90% death with strains CBS 114402, CBS 117900 and CBS 131838 (clinical, *C. carrionii*); CBS 131736 (environmental, *C. carrionii*) (log-rank, *p* < 0.001), and CBS 114406 (environmental, *C. yegresii*) (log-rank, *p* < 0.001). The most virulent strain was a soilborne isolate of *C. carrionii*, CBS 131736, where inocula of 10^4^–10^6^ conidia resulted in 100% death of *G. mellonella* larvae (logrank, *p* < 0.00) within 4–7*d*. By the time of death, larvae were deeply melanized as observed with other fungal pathogens (Cotter et al. 2000). An inoculum of 10^6^ resulted in 90% survival after injection of CBS 114405 and CBS 114407 (environmental, *C. yegresii*) and CBS 861.96 (environmental, *C. carrionii*) (log-rank, *p* > 0.05), and 80% survival with CBS 100434 (clinical, *C. carrionii*) (log-rank, *p* > 0.05). In seven of the nine strains tested, the survival rate obtained with inocula of 10^4^ did not differ significantly from the survival obtained in the PBS injected controls (log-rank, *p* > 0.05) except CBS 131736 (*p* < 0.001) and CBS 114406 (*p* < 0.01). To determine the influence of temperature, infection rates were compared at 27 °C and 37 °C; no statistically significant difference was obtained in the survival of *G. mellonella* larvae.

### Fungal morphology in hemolymph and histopathology

**Fig. 3:**
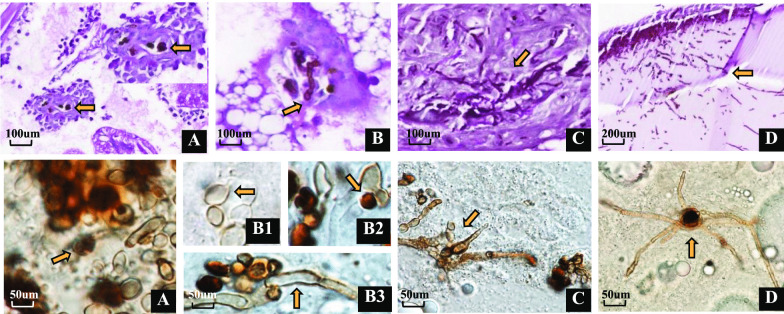
Histopathology with PAS staining (upper images **A**–**D**) and microscopy (lower images **A**–**D**) with hemolymph of *G. mellonella*. Larvae infected with *Cladophialophora carrionii* (clin., CBS 117900) at a dose of 10^5^ CFU per larva, incubated at 37 °C. **A** at day 1; **B** at day 3; **C**. at day 7; **D** at day 10, post infection respectively. **A**–**D** from dead larvae. In upper images: **A**–**C**, fat body, magnification 40×, Nodule formations contained conidia and hyphae, and usually isolated into in the fat body; **D** digestive tract, magnification 20×, The fungus showed a tropism for the intestinal tract; the presence of hyphae around the intestinal tract; In lower images: **A**–**D**, magnification 20×, hemolymph. Arrows indicated conidia/hyphae. Scale bar, 500 µm

**Fig. 4:**
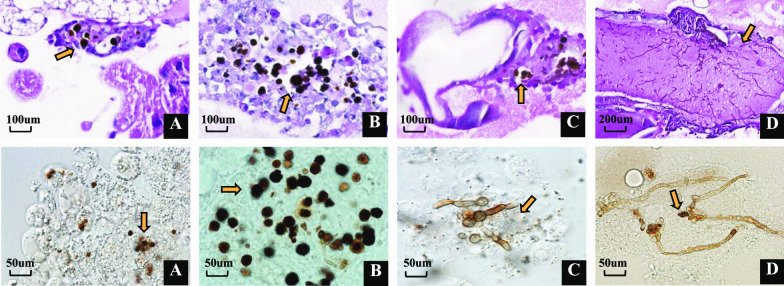
Differences in infection patterns between dead and living larvae by histopathology (PAS staining in upper images) and microscopy of hemolymph (in lower images) of *G. mellonella*. **A**–**B**: Larvae infected with *Cladophialophora carrionii* (CBS 861.96, envir.) at a dose of 10^5^ conidia / larva at day 10 post infection, In upper images; **A** living larvae;a few oenocytes present at fat body, magnification 40×; **B** dead larvae, oenocytes produced a lot at fat body, magnification 40×. **C**–**D**. Larvae infected with *C. carrionii* (CBS 117900, clin.) at a dose of 10^5^ conidia/larva at day 10 post infection; **C** living larvae; nodule formations contained conidia mainly and showed a tropism for tracheal system,magnification 40×, **D** dead larvae, hyphae were produced in significantly larger amounts, and showed a tropism for the intestinal tract, magnification 20×. In lower images: **A**–**D**, magnification 40×, hemolymph. Arrows indicated hemocytes /oenocytes /conidia/hyphae. Scale bar, 500 µm

Fungal distribution and morphology in the hemolymph of *G. mellonella*, and the host’s immune response were observed microscopically and using PAS staining. At day 1 after *G. mellonella* infection, hemocyte nodules were observed, some containing fungal cells in the fat body (Fig. 3A). At days 3, 7 and 10, various degrees of filamentation were observed in all strains. Nodule formations contained conidia and hyphae (Fig. 3B–C), frequently surrounding tracheae, but also intestinal walls (Fig. 3D). Invasive branched hyphal formation was noted in two strains (CBS 117900, CBS 114402, clinical, *C. carrionii*) (Fig. 3C–D). Fungal elements reached solid organs, with significant tropism for the digestive tract (Fig. 3D). The infection was highly destructive and induced an intense inflammatory response (Fig. 4B, D). In dead larvae, oenocytes were produced more abundantly (Fig. 4B) than in living larvae (Fig. 4A); hyphae were produced in significantly larger amounts (Fig. 4D) in dead larvae than in living larvae (Fig. 4C).

In the infection model with environmental, non-virulent *C. carrionii* strain CBS 861.98, both tissue invasion and inflammatory response were subjectively less than in larvae infected with highly virulent *C. carrionii* clinical strains CBS 117900 and environmental strain CBS 131736, even in surviving larvae of up to day 3–7 post infection. Less solid-organ invasion is seen, and fungal elements were frequently seen in subcuticular regions and fat bodies. Nodule formation was scattered and smaller than observed in strains CBS 117900 and CBS 131738, and no hyphae were found (Fig. 4).

### Antifungal susceptibility in vitro and in vivo

In vitro susceptibility test results of the nine strains are shown in Table 1. Six strains of *C. carrionii* had the following MIC ranges: AMB 0.5–8 mg/L, ITZ 0.016–0.125 mg/L, VOR 0.063–1 mg/L, POS 0.016–0.063 mg/L, TER 0.016–0.25 mg/L, and three strains of *C. yegresii* had MIC ranges: AMB 0.25–0.5 mg/L, ITZ 0.25–0.5 mg/L, VOR 0.063–0.125 mg/L, POS 0.125–0.125 mg/L, TER 0.063–0.063 mg/L.

**Fig. 5:**
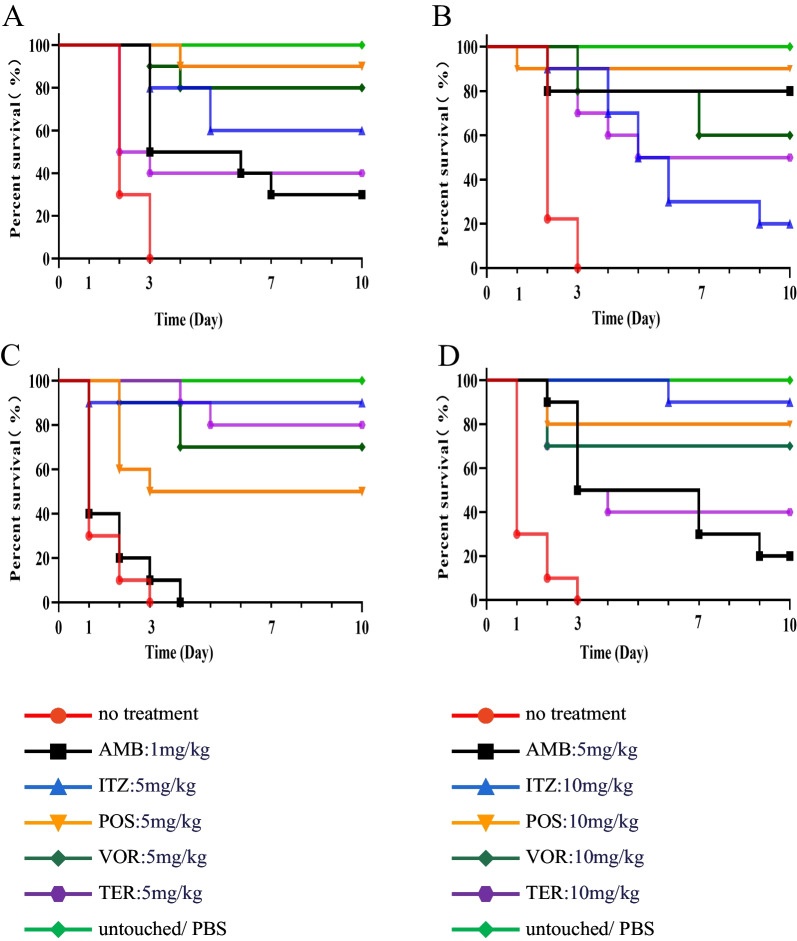
Survival curves of larvae infected with *Cladophialophora spp*. and treated with 5 selected antifungal agents. Left panels: AMB (amphotericin B) 1 mg/kg; ITZ (itraconazole), VOR (voriconazole), POS (posaconazole) and TER (terbinafine) all 5 mg/kg; Right panels: AMB 5 mg/kg and ITZ, VOR, POS and TER all 10 mg/kg. **A**–**B**: *Cladophialophora carrionii* (CBS 117900, clin, virulent) at a dose of 10^5^ conidia/larve; **C**–**D**: *Cladophialophora yegresii* (CBS 114406, envir, virulent) at a dose of 10^5^ conidia/larvae

In vivo, isolates CBS 117900 (clinical, virulent, *C. carrionii*) and CBS 114406 (environment, virulent, *C. yegresii*) were inoculated with 1 × 10^5^ conidia/larva, while larvae were treated with single doses of AMB (1 resp. 5 mg/kg), ITZ, VOR, POS and TER (all 5 resp. 10 mg/kg). Treated larvae were compared with control groups (the untouched group and PBS treated groups; Fig. 5). With strain CBS 117900 (Fig. 5A–B), protection was achieved with POS (5 and 10 mg/kg) and VOR (5 mg/kg) (logrank, *p* > 0.05). Itraconazole did not enhance larval survival with 10 mg/kg with control groups (logrank, *p* < 0.001), as only 10% larvae survived. AMB with 1 mg/kg yielded survival of 30% of larvae; with an increased dose to 5 mg/kg, 80% of larvae survived at day 10 (logrank, *p* > 0.05), however, TER (5 mg/kg and 10 mg/kg) yielded survival of 40–50% of larvae (at 5 mg/kg: logrank, *p* > 0.05; at 10 mg/kg: logrank, *p* < 0.01). With strain CBS 114406 (Fig. 5C–D), protection was achieved with ITZ (5 mg/kg and 10 mg/kg), VOR (5 and 10 mg/kg), POS (10 mg/kg) and TER (5 mg/kg) (logrank, *p* > 0.05). AMB did not or poorly enhance larval survival with treatment with 1 and 5 mg/kg (logrank, *p* < 0.001).

## Discussion

Chromoblastomycosis is a severe public health problem in hyperendemic regions such as Maranhão State in Brazil (Silva et al. 1995). Patients are prevalently agricultural workers with low socioeconomic status, who often are deprived from nearby medical assistance. When the infection has reached a severe state, effective management has become difficult. Prolonged therapy is necessary, often with limited clinical adherence and frequent relapses (Rasamoelina et al. 2020; Daboit et al. 2013). Given the scale of severe infections, with 1.47 cases/100,000 persons in some areas (Rasamoelina et al. 2020), the World Health Organization (WHO) has declared chromoblastomycosis as a neglected infectious disease. In clinical practice, despite the consistent presence of muriform cells in tissue, significant variation in appearance and severity is observed, which cannot always be explained by identity of the fungus or health status of the host. For understanding of this variation, knowledge on the mechanism and route of infection, and the natural behavior of etiologic agents are essential. When agents are traumatically inoculated from the environment, close relatives in *Fonsecaea* and *Cladophialophora* are likely to have an equal chance to cause disease. When particular species are more frequent in human tissue, these are also expected to be prevalent in the environment. With repeated isolation experiments, Vicente et al. (2008) yielded more unknown, strictly apathogenic species than species known from host. However, Costa et al. (2020) noted that the clinical species do commonly occur in environmental sources. Consequently, closely similar species show significant differences in growth and infective abilities.

In the present research on *C. carrionii* and its close relative *C. yegresii*, we observed that the above differences are not only between species, but also within the same species. Significant differences were found in growth velocity, melanization, virulence and antifungal susceptibility, which were not linked to taxonomic differences. An explanation why *C. yegresii* has not yet been found on humans may be its lower thermotolerance, the fungus being unable to grow at 37 °C, a property which seems to be species-specific. Infective ability in the *G. mellonella* larvae, conducted at lower temperature, is nevertheless present in one out of three strains. *C. carrionii* environmental strain CBS 131736 yielded highest killing rates, and had most intense melanization and highest growth speed, while another environmental strain of the same species, CBS 861.96 with no virulence had lowest melanization and grew rather slowly.

Larvae of *G. mellonella* inoculated with *Cladophialophora* and controls were processed by hemolymph collection (squeezing) and whole-larva embedding (sagittal sectioning) to evaluate morphological aspects of host–pathogen interactions (Fig. 3). The progress of infection was monitored after 24 h (A), 3 days (B), 7 days (C) and 10 days (D). *G. mellonella* induces an early immune response at 24 h after inoculation, with appearance of nodules that contain single cells only (Fig. 3A). From 3 days post inoculation, nodules contain both single cells and hyphae (Fig. 3B–D). Nodules increased in number and in dimension, depending on biological characteristics of the *Cladophialophora* strain. This feature was particularly notable in larvae infected with CBS 17900 and CBS 114406 (high virulence strains), where pronounced filamentation resulted in large hyphal aggregates interfering with phagocytosis (Fig. 3C). We observed tropism of the fungus around the aero-digestive tract by day 10 (Fig. 3D). In larvae infected with CBS 861.96 (low virulence strain), a small number of hemocytes was present in the subcuticular area, in the hemolymph, and in close association with the aero-digestive tract, with nodules containing single cells only (Fig. 4A). In healthy larvae, oenocytoids, which represent 5–10% of the total hemocytes, contain cytoplasmic phenoloxidase and participate in the melanization of the hemolymph (Altincicek et al. 2008). Oenocytes can also secrete nucleic acids that have been described as “a new alarm signal” in the defense of these insects (Trevijano-Contador et al. 2019).

In infection sites of dead larvae, hemocytes formed multicellular aggregates containing much more oenocytoids than those in living larvae (Fig. 4A–B); this corresponds with the observation that an exaggerated immune response tends to be lethal for the larvae (Lu et al. 2014).

Several studies (Aufauvre-Brown et al. 1998; Fromtling et al. 1989) have suggested that environmental and clinical strains of *Aspergillus fumigatus* might differ in their virulence potential (Cheema & Christians 2011). In our study, differences in pathogenicity could not be linked to origins of strains, either clinical or environmental. These findings are in disagreement with previous murine experiments with *C. carrionii,* where two isolates from patients exhibited invasive abilities while an environmental isolate failed to produce lesions in mice (Yegres et al. 1991).

All nine isolates proved to be susceptible to itraconazole, voriconazole, posaconazole and terbinafine. Amphotericin B was less active, which agreed with previous studies (Vitale et al. 2009; Deng et al. 2013). Two strains had relatively high virulence in vivo: clinical isolate *C. carrionii* CBS 117900, and environmental isolate *C. yegresii* CBS 114406. The protective effect of itraconazole in these two strains appeared entirely different. Even the three strains of *C. yegresii*, which were all isolated from *Sterinocerus* cactus thorns in a limited area of Falcón state in Venezuela, showed large differences in invasive potential. The observed clinical differences in CBM provoked by a single species may thus partly be due to significant differences between individual strains. Species affiliation thus appears to be poorly predictive for clinical appearance and course of infection. It has long been supposed, that patients with CBM are otherwise healthy and immunocompetent. Interestingly, a significant share of patients with severe CBM had an as yet undescribed mutation (Sobianski Herman et al. 2023) in the Caspase recruitment domain-containing protein 9 (*CARD9*), a rare inherited immune disorder.

Regarding limitations that should be considered in this study are that we did not statistically quantify the associations between the fungus and cells type presented in these tissues.

## Conclusion

Clinical variation is an interplay of the abilities of the coincidentally inoculated potential etiologic agent of disease, and the genetic condition of the human host. More detailed analysis of these adverse parameters may lead to personalized precision medicine to mitigate this severe and recalcitrant disease. Studies with the *G. mellonella* model are instrumental in understanding variable pathogenesis and in vivo efficacy of antifungal agents.

### Supplementary Information

**Additional file 1**. **Fig. S1** Growth speed on different medium of nine strains of *Cladophialophora* spp., incubated at 25 °C for 2 weeks. Note: OA: Oatmeal agar; PDA: Potato Dextrose agar; MEA: 2% Malt Extract Agar

**Additional file 2**. **Table S1** Single dose of each antifungal drug injected into larvae.

## Data Availability

Not applicable.

## Abbreviations

WHO: World Health Organization
CBM: Chromoblastomycosis
OA: Oatmeal agar
MEA: Malt extract agar
PDA: Potato dextrose agar
PBS: Phosphate-buffered saline
MIC: Minimum inhibitory concentrations
AmB: Amphotericin B
ITZ: Itraconazole
VOR: Voriconazole
POS: Posaconazole
TER: Terbinafine
