# Supplementary figures and images for: Galleria mellonella in vitro model for chromoblastomycosis shows large differences in virulence between isolates
Source: IMA Fungus. 2024 Mar 8;15:5. doi: 10.1186/s43008-023-00134-5 (PMC10921731; doi:10.1186/s43008-023-00134-5)

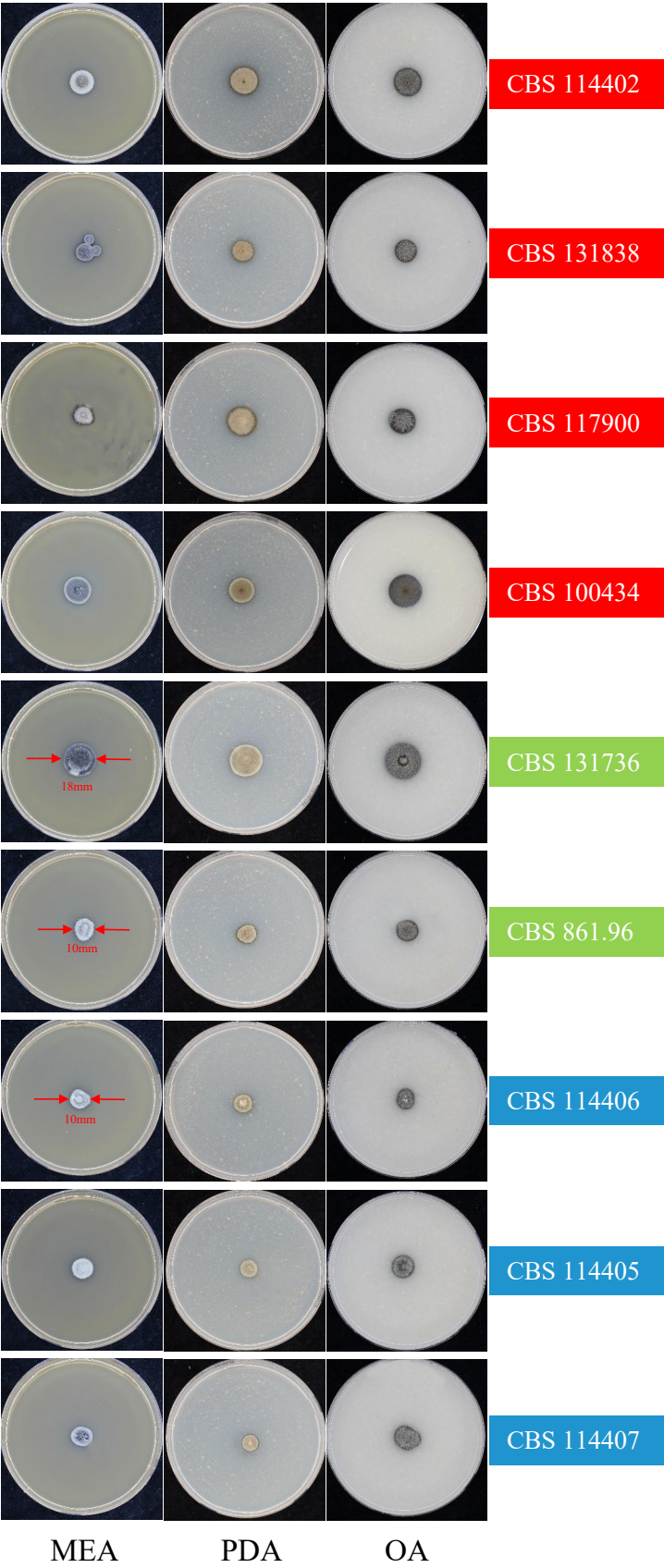

Supplement: Supplementary file 1 — Additional file 1. Fig. S1 Growth speed on different medium of nine strains of Cladophialophora spp., incubated at 25 °C for 2 weeks. Note: OA: Oatmeal agar; PDA: Potato Dextrose agar; MEA: 2% Malt Extract Agar [file 43008_2023_134_MOESM1_ESM.pdf]
