# Supplementary material for: Galleria mellonella in vitro model for chromoblastomycosis shows large differences in virulence between isolates
Source: IMA Fungus. 2024 Mar 8;15:5. doi: 10.1186/s43008-023-00134-5 (PMC10921731; doi:10.1186/s43008-023-00134-5)
Supplement: Supplementary file 2 — Additional file 2. Table S1 Single dose of each antifungal drug injected into larvae. [file 43008_2023_134_MOESM2_ESM.docx]

| Antifungal drugs | Drug Concentration (mg/kg) | |
| --- | --- | --- |
| Amphotericin B | 1 | 5 |
| Voriconazole | 5 | 10 |
| Itraconazole | 5 | 10 |
| Posaconazole | 5 | 10 |
| Terbinafine | 5 | 10 |
